# Assessing the RNA integrity in dry seeds collected from diverse endangered species native to the USA

**DOI:** 10.3389/fpls.2025.1585631

**Published:** 2025-05-13

**Authors:** Hannah M. Tetreault, Zoe Zingerman, Lisa Hill, Shaimaa Ibrahim, Joyce Maschinski, Katherine D. Heineman, Christina Walters

**Affiliations:** ^1^ United States Department of Agriculture, Agricultural Research Service (USDA-ARS) National Laboratory for Genetic Resources Preservation, Fort Collins, CO, United States; ^2^ Center for Plant Conservation, Escondido, CA, United States; ^3^ San Diego Zoo Wildlife Alliance, Conservation Science, Escondido, CA, United States

**Keywords:** RNA integrity, seed quality, seed aging, native seeds, genebank, wild plant populations, viability

## Abstract

**Introduction:**

Dry seeds do not show obvious signs of life and so testing for viability, health and life expectancy can be challenging. Usually testing seed quality involves adding water and measuring metabolic capacity or growth potential by vital staining or germination assays. Importantly, most laboratory seed tests are intended to assay immediate viability, while most genebanks need tests that predict seed performance in the distant future. All currently available assays require considerable a priori knowledge of germination conditions and seeds large enough to dissect. Germination conditions are often unknown for seeds produced from wild species and are an important criterion for seed testing.

**Methods:**

Using standardized methods (i.e., commercially available kits) we tested the feasibility of adapting a new seed quality assay that measures RNA integrity and is promising for cultivated species, to seeds from wild species. Most of the 100 wild species we include are rare or endangered and in need of preservation through genebanking. To determine the feasibility of measuring RNA integrity in seeds from wild populations, we compared the quality of RNA extracted from seeds that were recently harvested to those of the same species that have been genebanked for 16 to 41 years, with various seed traits examined for interference with RNA extraction and characterization.

**Results:**

We demonstrate reliable characterization of RNA quality across a diverse group of plants, despite variation in germination requirements, seed morphology or composition. RIN (RNA Integrity Number) values were usually high across all samples and variables, attesting to both the quality of newly collected material as well as its maintenance under genebanking conditions. This study conclusively demonstrates the feasibility of reliably extracting and characterizing RNA from dry seeds collected from wild populations, regardless of a variety of seed traits and morphologies. Relationships between RNA quality and seed age and viability require further exploration.

## Introduction

Germination tests are the “gold standard” to evaluate seed health. Seeds from most domesticated species germinate within a few days after sowing and so laboratory germination tests can quickly and reliably assess viability, normal growth and germination speed (AOSA, 2019). Laboratory tests are more labor intensive and less informative for seeds of many wild species, which tend to germinate slowly (weeks to years) and asynchronously, have high interspecific variability (Hamasha and Hensen, 2009; Zhang et al., 2020) and complex dormancy status (Kildisheva et al., 2019; Pedrini and Dixon, 2020; Baskin and Baskin, 2021). Unlike crop species, many wild plant species display some degree of seed dormancy. Seed dormancy is a natural mechanism that regulates germination through various physical or physiological means imposed by the seed coat or within the embryo (Baskin and Baskin, 2000). Treatments to stimulate germination of seeds from wild species, at species and population-specific levels, are needed to increase testing reliability and increased stand establishment for wild plants used as genetic resources, restoration or evolutionary biology questions (Etterson et al., 2016; Coyne et al., 2020). In many seed testing labs, time allowed to complete a germination assay is too short for wild-collected samples and the assay is prematurely terminated by testing viability of ungerminated seeds using a vital stain, such as tetrazolium that indicates metabolic or respiratory capacity (Miller, 2005; França-Neto and Krzyzanowski, 2019).

Plant genebanks, commonly referred to as “seed banks,” use seed testing as a standard operation to assess initial quality, develop procedures for recovery and growth, and ensure that germination capacity and genetic integrity of the stored material is maintained (Hay and Probert, 2013; FAO, 2018; De Vitis et al., 2020). Despite cold, dry storage conditions used to prolong viability of seeds maintained in genebanks indefinitely, aging is inevitable (Walters et al., 2010; Hay et al., 2022). A genebank needs to know the sample’s ‘expiration date’ so that it can be used or replaced before its utility is compromised. Hence, the design of laboratory tests used by genebanks must accommodate comparisons across time, with sufficient statistical power to detect small change that occurs over decades. Germination assays may not be efficient or effective at detecting subtle changes of a stored sample. Germination assays provide categorical (nominal) dichotomous data (i.e., normal/abnormal or alive/dead), requiring large sample sizes to detect minor increases in mortality (Tetreault et al., 2023). High variability among germination tests of wild-collected seeds confounds comparisons separated by time. Also, repeated testing depletes the sample – a critical problem when genebanking germplasm from small or remote populations having low fecundity. Moreover, the nature of dichotomous designation of alive or dead does not reveal nonlethal changes that precede mortality. Because they are the culmination of aging, lost germination capacity or mortality are particularly insensitive symptoms of aging. Assays that mark deteriorative progress toward a mortality threshold would better serve genebank needs.

RNA integrity is an emerging technique that detects degradation in seeds from domesticated species before large scale mortality occurs (Fleming et al., 2017, 2019; Walters et al., 2020; Zhao et al., 2020; Tetreault et al., 2023). The assay relies on RNA that accumulates during seed maturation and slowly degrades in dry storage because RNAases appear inactive in dry cytoplasm (Spanò et al., 2007). Non-enzymatic oxidation of RNA over time leads to steadily increasing fragmentation that is visible during electrophoresis, especially in the 25S and 18S rRNA fractions (Brocklehurst and Fraser, 1980; Kranner et al., 2011; Fleming et al., 2019; Walters et al., 2020). Damage to DNA structure occurs as well; however DNA is a more stable molecule than RNA and does not appear to fragment until long after death (Walters et al., 2006). The extent of RNA fragmentation can be quantified by RIN (RNA Integrity Number), which considers the relative size of both rRNA peaks and other features from the electropherogram. RIN is now an industry standard to characterize RNA quality because it is reliable, reproducible and easily standardized; the formula calculating RIN is proprietary (Mueller et al., 2004; Schroeder et al., 2006). In dry seeds, RIN appears to function like a clock that ticks at different rates depending on how fast seeds age. Hence, RIN declines linearly with time in contrast to the time courses for viability loss which are sigmoidal (Fleming et al., 2019; Walters et al., 2020; Tetreault et al., 2023). Before now, application of RIN to seed aging has only been reported using domesticated seeds that were included in legacy collections with highly controlled provenance.

Using RIN to detect seed aging offers advantages of a standardized procedure that detects early stages of degradation that is independent of seed dormancy. Adapting RIN assessments for use in genebanks will require general knowledge of the applicability of these assays to diverse species. Importantly, RIN decline over time should reflect aging rate or longevity, with kinetics relatable to sample expiration dates. This is an unusual application of RIN, which is normally used to confirm that an extraction yields RNA of sufficient quality for sequencing; a low RIN is mostly interpreted as a flawed extraction. In this proposed new application, observed differences of RIN in extractions of seeds separated by storage time could either reflect artifacts created during extraction or electrophoresis. Alternatively, low RIN may be the desired signal of seed aging. To determine the risk of artifact, we have aimed to determine the contribution of storage time to RIN assessments in seeds, relative to other factors that contribute to variation of RNA quantity and quality, such as diverse physiologies or morphologies as well as RIN assay uncertainty.

In this work, we compared RNA quality of paired seed cohorts of over 100 diverse wild plant populations harvested from the same locales after 2021 and before 2005 to allow paired comparisons of RNA extraction and characterization parameters. The seeds were from wild populations, predominantly rare or endangered species, native to the US representing diverse botanical families, geographic origins, seed morphologies, chemical compositions, and physiologies. We evaluated whether 1) RIN was lower in the stored seeds compared to the newly harvested ones; 2) low concentration or purity of extracted RNA contributed to low RIN values or anomalous results; 3) particular seed traits interfered with RNA extraction or characterization; and 4) anomalous results could be resolved by additional tests.

## Materials and methods

### Seed material

Seeds from approximately 100 native plant species of conservation interest were identified, and collections were obtained from the same population at two time points, referred to as “recently harvested” (sampled between 2021 and 2024) and “stored” (sampled before 2005, with mean harvest year occurring in 1995 ± 6 yrs) (**Table 1**). An “accession” refers to a seed sample of a particular species from a particular sampling year (i.e., cohort). The harvest year for a cohort is denoted with an ‘H’ after the year; for example, seeds collected in 2001 will be identified as 2001H. Storage conditions mostly reflected international recommendations for genebanking (low RH and -18˚C) (FAO, 2018), though some seeds were placed under refrigerated (5˚C) or cryogenic (~ -180˚C) conditions. Seeds were stored either at botanical gardens within the Center for Plant Conservation (CPC) network or at the National Laboratory for Genetic Resources and Preservation in Fort Collins, CO. Analyses used a bulk sample consisting of seeds combined from several maternal lines.

**Table 1:** The number of species, harvest year and number of replications/species included in the large study.

Factor	Average ± std dev or count	
	Recently harvested	Stored
# species	103	105
harvest year	2022 ± 1	1995 ± 6
# replications/species	4.8 ± 2.0	4.9 ± 2.7
Nanopore diagnostics		
[RNA] (ng/µl)	202 ± 185	194 ± 187
260/280	2.01 ± 0.16	1.91 ± 1.44
260/230	1.67 ± 0.60	1.61 ± 0.65
Agilent bioanalyzer diagnostics and data		
RNA area	388 ± 188	382 ± 203
[RNA] (pg/µl)	2252 ± 959	2496 ± 1862
rRNA ratio	2.07 ± 1.09	1.82 ± 1.53
RIN	7.7 ± 1.3	6.8 ± 1.7
# electrophoresis runs	685	655
# suspect	45	44

Summary of Nanophore diagnostics gathered from the spectrophotometer on RNA purity and on fragmentation of RNA from the Agilent bioanalyzer.

### Sample preparation

Samples were prepared to address how variation in seed size, embryo presence (i.e., seed fill), thick or water-impermeable seed coverings (i.e., hard seeds), and seed maturity, type of tissue and lipid content affect RNA quality and RIN values. Between 1 and 66 mg (average of 12.3 ± 6.2 mg) of seed tissue were used per biological replicate in RNA extractions. The median number of seeds used in an extraction was 13, but as many as 500 seeds were used in a single extraction for the smallest seeds (e.g. *Kalmiopsis fragrans*, 0.012 mg/seed; *Leiophyllum buxifolium*, 0.025 mg/seed). All efforts were made to use a clean seed sample that lacked plant debris. The number of biological replicates per accession ranged from 1 to 16 (average of 5.6 ± 3.2). For accessions with few or tiny seeds we used fewer biological replicates. Because it is possible that RNA yield in a sample could have been affected by non-seed material (e.g., chaff, flower parts), we explored the quantity of RNA yield and subsequent RIN score in seed parts (seed coats, embryos, cotyledons) and in samples cleaned to varying degrees.

#### Seeds lacking embryos

Seeds harvested from wild populations often have outer coverings that appear normal, but there is no embryo inside. The incidence of “empty” or “unfilled” seeds in this study varied among species and cohorts from 0 to 99%, with an average of 20% (Walters et al., unpublished). We reasoned that seeds with and without embryos may have different RNA properties and that the inability of empty seeds to germinate would confound comparisons of RIN with germination tests. For this reason, empty seeds were excluded from RNA extractions when an accession presented greater than 20% empty seeds and the mass per seed was greater than 0.2 mg. Sorting filled from empty seeds involved the laborious step of dissecting seeds under a microscope, carefully pulling out filled seed. To test if this step affected RNA yield, purity or RIN, we identified a subset of 15 accessions having greater than 35% empty seeds and compared RNA parameters for samples in which the empty seeds were and were not removed prior to RNA extraction.

#### Seeds with thick outer coverings

Many of the seeds in this study set were covered by thick layers of maternal tissues such as remnant fruit or floral parts, or had thick, probably water-impermeable seed coats (e.g., seeds from Fabaceae). In some cases, seed cleaners at botanical gardens removed maternal tissues from one of the cohorts but not the other. To be consistent between cohort pairs, the same tissues were used in RNA extractions, which sometimes required removing pods or external layers (e.g., *Amorpha herbacea* var. *crenulata* (2003H), *Hymenoxys texana* (2005H) and *Osteomeles anthyllidifolia* (2021H and 2000H)). For very large seeds, it made sense to use only the embryo which could be dissected out and used for RNA extraction (e.g., *Arctostaphylos catalinae* (79 mg/seed), *Castela emoryi* (36 mg/seed), *Rhus kearneyi* spp. *kearneyi* (45 mg/seed) and *Ziziphus celata* (511 mg/seed)). Apart from embryo-only samples, between 3 and 85% (average of 40%) of the grain mass of seeds was removed by careful peeling, cutting or scraping under a microscope in seeds that weighed more than 0.3 mg and had apparently thick outer layers. To test if this affected RNA yield, purity or RIN, we identified a subset of 28 accessions having less than 25% empty seeds and compared RNA parameters for samples in which outer coverings were and were not removed prior to RNA extraction.

#### Seed maturity

Seeds from wild populations typically do not mature synchronously and so there can be a range of maturities within an accession. Most of the accessions in this study appeared to contain seeds that were fully mature with appropriately dark coloring and low incidence of shriveling. However, there were four accessions that presented within-sample variation in coloring reminiscent of slight variation in seed maturity at harvest (**Supplementary Figure 1**). To test if this apparent difference in seed maturity affected RNA yield, purity or RIN, we compared RNA parameters for replicates prepared using seeds having only dark or light coloring.

#### Tissue type

To examine if tissue types and relative contribution of tissue mass to seed size impacted RNA yield, purity or RIN, we dissected embryonic and nutritive tissues from *Abies fraseri* (both cohorts), *Amaranthus pumilus* (1987H) *Astragalus magdalenae* var. *peirsonii* (2021H), *Lupinus westianus* var. *aridorum* (2021H), and *Rhus kearneyi* ssp. *kearneyi* (both cohorts) and compared RNA parameters among the different tissues.

#### Lipid content

The amount of lipid in seeds was inferred from the enthalpy of lipid melting transitions, which was measured in a separate study using differential scanning calorimetry (DSC) (Walters et al., unpublished). Briefly, samples were cooled and warmed to and from -150˚C at 10˚C/min and enthalpy was calculated by the size of melting transition during heating. A melting enthalpy of 60 J/g oil was used to translate melting enthalpy based on sample mass to g lipid per g of seed. To relate RNA yield, purity or RIN to the lipid content of seeds within the dataset we measured melting enthalpy from known seed mass.

#### RNA extraction and integrity assays

RNA was extracted from seed samples prepared as described above using approximately 10 mg of dry tissue per sample. The sample was prepared by combining the dry tissue and ~1mg of polyvinylpyrrolidone-40 (PVP; Fisher Scientific, Fair Lawn, NJ) in a 2 ml test tube with a nickel/lead steel shot bead (Ballistic, Inc., Hamel, MN), then flash frozen in liquid nitrogen before using the TissueLyser ll (Qiagen, Hilden, Germany) to grind tissue to a fine powder. For RNA isolation, the Qiagen Plant RNeasy kit (Qiagen, Hilden, Germany) and Takara Nucleospin RNA kit with Fruit-mate (Takara, Düren, Germany) were used per the manufacturer’s instructions. The DeNovix DS-11 FX+ Spectrophotometer (DeNovix, Wilmington, DE) was used to determine RNA yield and purity. A subsample was used immediately for electrophoresis and the remaining sample was archived at -80˚C.

Subsamples used for electrophoresis were diluted to 2 ng µL^-1^ in nuclease-free water and electrophoresed using the Agilent Bioanalyzer (Agilent, Waldbronn, Germany) with Agilent RNA 6000 Pico chips and the Plant RNA Pico assay (Agilent 2100 Expert software version B.0208.SI648 R3) per the manufacturer’s protocol. RNA concentration, rRNA ratio and RIN were quantified using Agilent RNA 6000 Pico chips and the Agilent 2100 Expert software which analyzes peak and fragment sizes and calculates RIN using a proprietary formula (Fleming et al., 2017; Tetreault et al., 2023).

The ratios of absorbance at 260 and 280 nm and at 260 and 230 nm were used to assess the purity of extracted RNA. Ratios near 2.0 reflect a successful extraction with relatively pure RNA. A 260/280 ratio near 1.7 indicates higher than desired levels of DNA and absorbance ratios less than 1.7 usually indicate contamination by proteins, polysaccharides or reagents used during extraction.

#### Statistical analysis

Analysis of variance tests were done within species to determine significant difference in RIN when special treatments were applied to each species. Analysis of variance tests were calculated using JMP 12.2.0 (SAS Institute Inc., 2015).

## Results

### Factors associated with reliable electrophoresis

**Figure 1:**
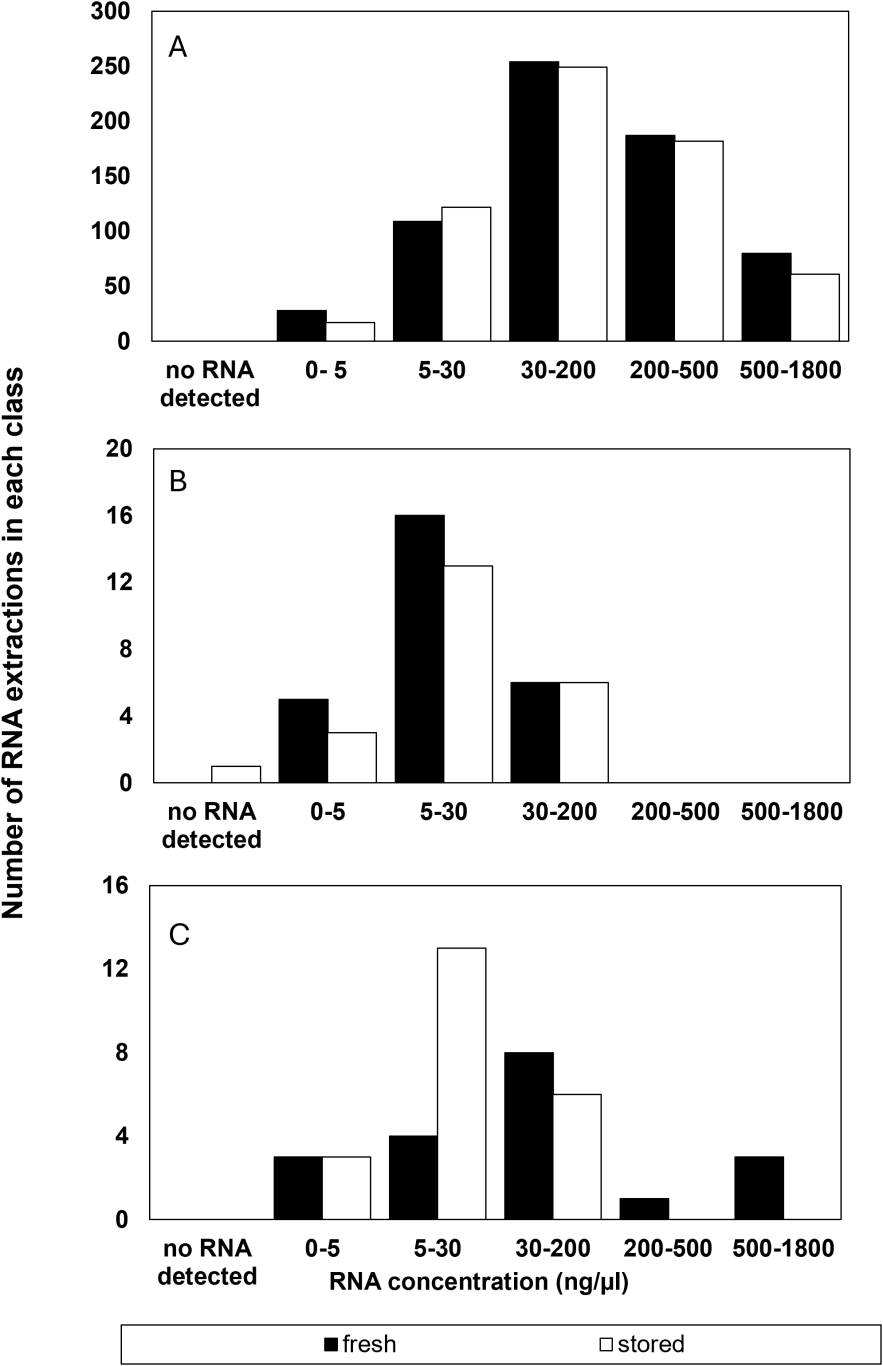
Number of RNA extractions in each class of RNA concentrations (ng µl^-1^) that **(A)** were considered successful and included as RIN data or **(B)** had a suspect RIN value in the first electrophoresis run however provided a reliable RIN value after electrophoresis was repeated, and **(C)** the number of samples that were considered to give an unreliable RIN value after reanalysis from a failed first run.

RNA was extracted from over 200 accessions of wild seeds from diverse species that were harvested recently (103 species) or stored for at least 15 years under genebanking conditions (105 species). More than 1200 extractions were performed and characterized with over 1350 electrophoresis runs (**Table 1**). On average, 4.8 (± 2.0) and 4.9 (± 2.7) RNA extractions were performed per species for recently harvested and stored treatments, respectively. A RIN result was deemed reliable if its value was within 2 units of the average of 3-4 replicate runs for the accession. Overall, 96% of the RNA extractions provided reliable RIN values on the first or second electrophoresis run (**Figure 1A**).

Most (93%) of all RNA extractions provided a reliable RIN value on the first electrophoresis run using the bioanalyzer (**Figure 1A**). Experimental error was initially apparent in about 7% of the electrophoresis runs or 79 of the 1210 RNA extractions (**Figure 1B**). Electrophoresis “failed” in a total of 87 of 1365 electrophoresis runs, in that either no RIN value was calculated (37 runs) or the calculated RIN was inconsistent with the average determined for the accession (50 runs). Most of the 37 runs in which the Agilent software failed to calculate RIN were due to mis-calling the location of the 25S and 18S rRNA peaks. Thus, about 3% of all electrophoresis runs resulted in no RIN data (**Figure 1C**). That did not affect overall RIN averages, but did risk further depletion of the accession by requiring another replicate. We re-ran electrophoresis in 26 of the 37 original extractions from samples that had been archived at -80˚C and found that the problem was resolved in all but 4 samples. Anomalously low (48 runs) or high (2 runs) RIN values were obtained in 50 of the 87 “failed” electrophoresis runs (or 4% of all runs) and there is a risk that these might be attributed to seed quality rather than an extraction or electrophoresis mishap. We tested the possibility of an error during electrophoresis by re-running 36 of the 50 original extractions from samples that had been archived at -80˚C and found that the problem was resolved in all but 4 samples. In summary, of the 87 electrophoresis runs initially deemed to provide unreliable or no RIN data, 54 could be resolved by re-running an archived sample, 23 were not re-run, and 8 RIN outcomes reflected a problematic extraction. From this analysis of experimental error, we concluded that, in our hands, about 1% of RIN determinations may falsely indicate a deteriorated sample and about 0.5% of extractions may not provide a RIN measurement. Also, at least 60% of detected errors could be corrected by repeating electrophoresis a second time.

We examined spectroscopic properties of RNA extractions to determine whether RNA concentration or purity contributed to failed or suspect electrophoresis runs that gave either no or anomalous RIN values. RNA concentration fell within the 25 to 500 ng/µl range recommended by the manufacturer for RNA integrity characterization (Schroeder et al., 2006) for most of the extractions (874 out of 1210), with 129 and 206 extractions giving RNA concentrations above or below, respectively, the manufacturer’s guidelines (**Figure 1A** and **Table 1**). There was about a 3% failure rate (incidence of no or anomalous RIN values) in extracts at or above the recommended RNA concentration and over 60% of these could be resolved by a second electrophoresis run of the same extract. In contrast, the failure rate in extracts containing less than 25 ng/µl was 28% and only about ⅓ could be resolved with a second electrophoresis run. In other words, roughly ¾ of failed electrophoresis runs in this study can be attributed to RNA concentrations that were too low (**Figure 1C**). Notably, valid RIN assessments were obtained in extracts despite extremely low RNA concentrations of 0-5 ng/µl range (**Figure 1A** and 1B). There appeared to be no difference between RNA concentration and failed electrophoresis runs in recently harvested and stored seeds. There was only one extraction in which RNA was not detected by absorbance at 260nm (a stored accession of *Remirea maritima* (Cyperaceae) in which all biological replicates contained less than 10 ng/µl RNA despite being cleaned to remove most of the empty seeds).

**Figure 2:**
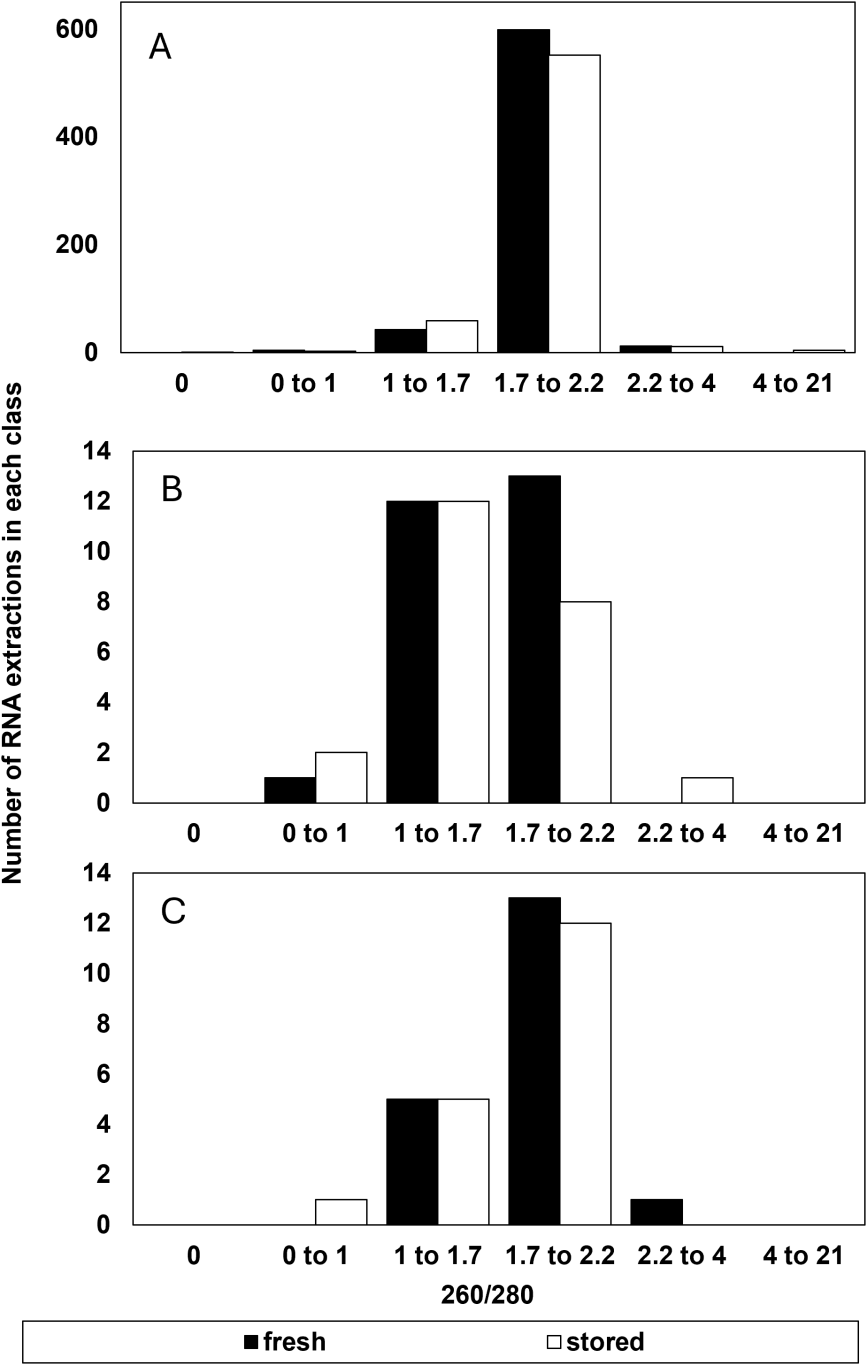
Number of RNA extractions in each class of 260/280 ratio that **(A)** were considered successful and included as RIN data or **(B)** had a suspect RIN value in the first electrophoresis run then provided a reliable RIN value after electrophoresis was repeated, and **(C)** the number of samples that were considered to give an unreliable RIN value after reanalysis from a failed first run. A reliable RIN value has a 260/280 = 2.0.

**Figure 3:**
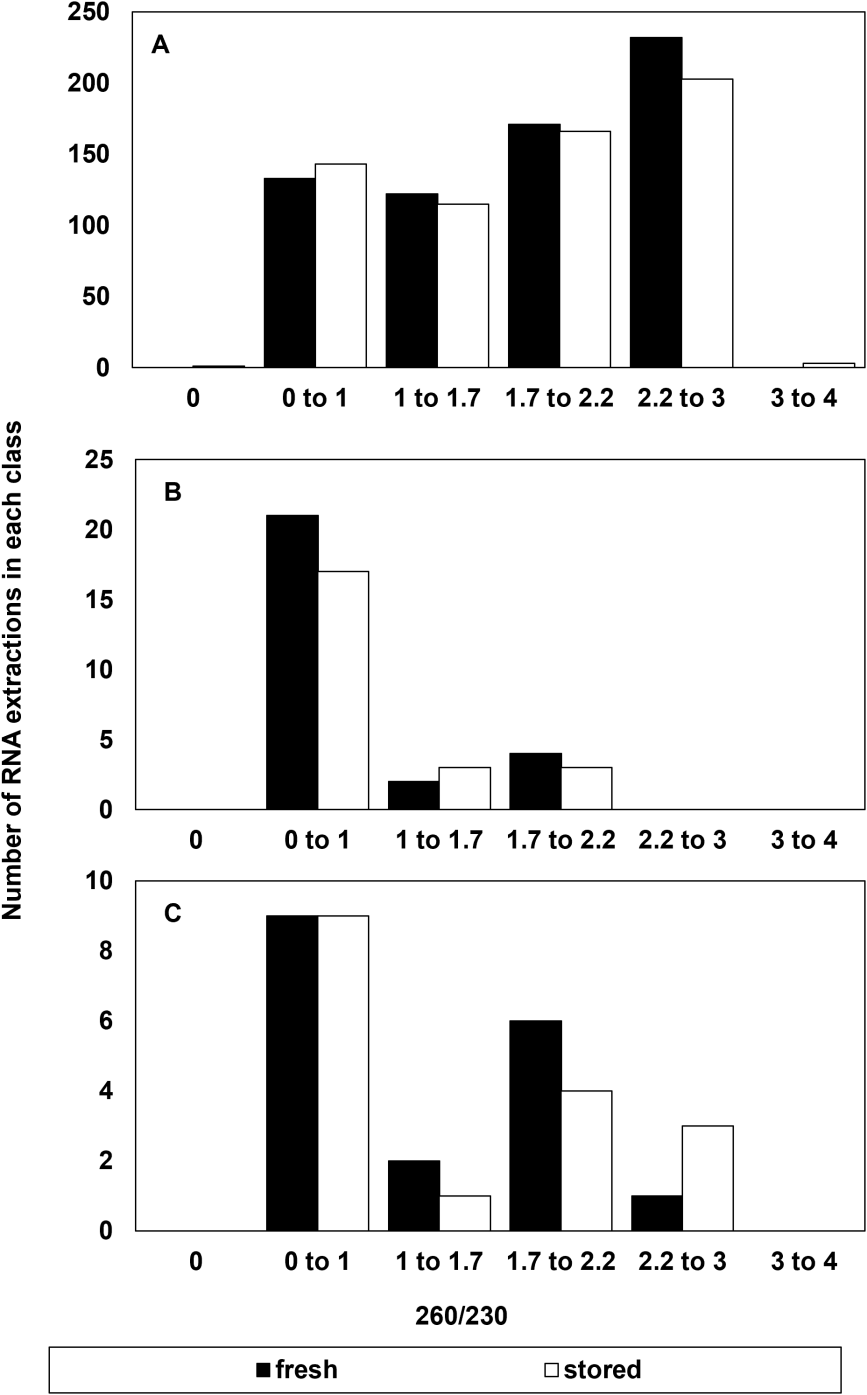
Number of RNA extractions in each class of 260/230 ratio that **(A)** were considered successful and included as RIN data or **(B)** had a suspect RIN value in the first electrophoresis run then provided a reliable RIN value after electrophoresis was repeated, and **(C)** the number of samples that were considered to give an unreliable RIN value after reanalysis from a failed first run.

There was little indication that RNA purity, indicated by absorbance ratios at 260 and 280nm or 260 and 230 nm contributed to the reliability of RIN determinations (**Figures 2**, **3**). The majority of extracts (1094 out of 1210) had A260/280 ratios between 1.7 to 2.2 indicating relatively high purity without protein or polysaccharide/phenolic contaminants (**Figures 2A, B**). About 4% of these failed to give reliable RIN results (**Figure 2A**) and half of these also had low RNA concentrations (< 30 ng/µl) (not shown). There were 116 (of 1210) extractions that gave A260/280 ratios outside the recommended 1.7 to 2.2 range. Of these, 80% also had low RNA concentration (< 30 ng/µl) and 32% failed to give reliable RIN results (**Figure 2C**). In other words, low RNA concentration was also a common factor in over 90% of extracts with low purity based on A260/280 ratios. A secondary marker of RNA purity, the ratio of absorbance at 260 and 230nm (e.g., A260/230), indicated the general presence of contaminants in extracts with a relatively broad distribution of samples giving A260/230 between 0 and 3 (**Figures 3A, B**). The highest proportion of failed electrophoresis occurred in samples with A260/230 ratios in the 0 to 1 range (**Figure 3C**). Over 80% of these extracts also had low RNA concentrations (< 30 ng/µl) and about 60% also had A260/280 ratios less than 1.7 (not shown). This suggests that contamination may interfere with reliable RIN determinations, but that low RNA concentration is also associated with low purity.

**Figure 4:**
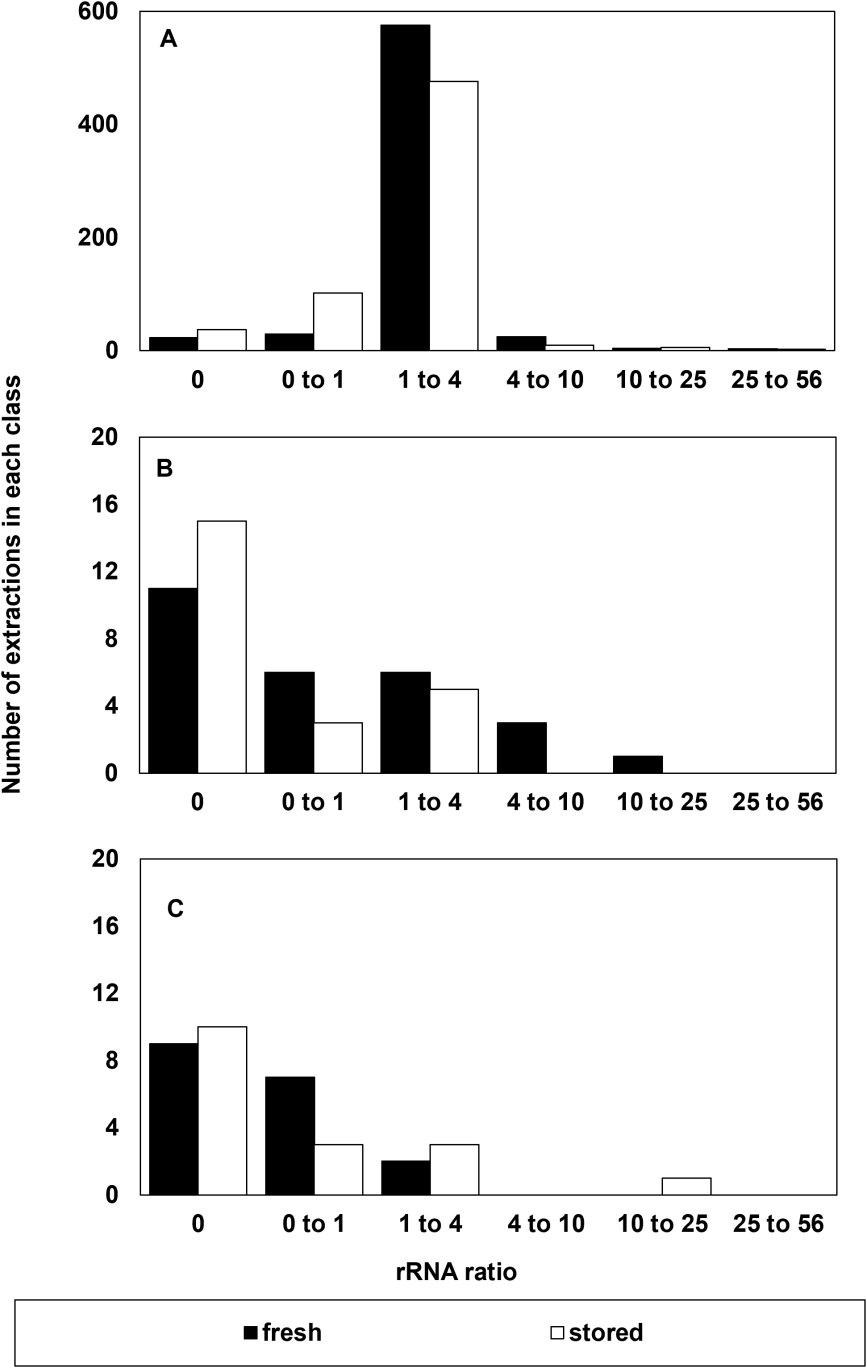
Number of extractions in each class of rRNA ratio that **(A)** were considered successful and included as RIN data, **(B)** had a suspect RIN value in the first electrophoresis run then provided a reliable RIN value after electrophoresis was repeated, and **(C)** the number of samples that were considered to give an unreliable RIN value after reanalysis from a failed first run. A reliable rRNA ratio is between 1 and 4.

Peaks representing elution of ribosomal RNA (25S and 18S) dominate electropherograms when RNA is intact because rRNAs are the most abundant, as well as largest, RNAs in the cytoplasm (Leaver, 2018). Hence, rRNA peaks have historically been used to evaluate RNA quality (Schroeder et al., 2006). In high quality RNA, the ratio of electropherogram peak areas for the 25S and 18S rRNAs should be about 2, and this ratio should decline as RNA fragments. In this study set, the rRNA ratio was between 1 and 4 in 1051 out of 1210 RNA extractions performed. The rRNA ratio was higher and lower than this range in 41 and 145 extractions, respectively (**Figure 4**). The majority (1003 or 98%) of 1051 extracts with rRNA ratios between 1 and 4 gave reliable RIN determinations (**Figures 4A, B**). Low RNA concentration (< 30 ng/µl) was noted in all failed electrophoresis runs when rRNA ratio >1 (18 extracts) and more than half of the failed electrophoresis runs when rRNA ratio <1 (42 extracts). From this assessment, the occurrence of extracted samples having both RNA concentration less than 30 ng/µl and rRNA ratios less than 1 is rare (~5%), but the occurrence of RIN anomalies (71%) is highest in this fraction. In contrast, the fraction of extracted samples having both RNA concentrations greater than 30 ng/µl and rRNA ratios greater than 1 is largest (73%), and the occurrence of RIN anomalies within this fraction is minor (0.5%).

Unlike the spectroscopic parameters measuring RNA concentration and purity (**Figures 1**-**3**), accession age (i.e., recently harvested vs. stored) has a significant effect on measured rRNA ratios (**Figure 4A**; *p* = 0.05). There are more accessions from the stored cohort that have rRNA ratios < 1 compared to counterparts that were recently harvested (*p* = 0.0014). Importantly, the incidence of RIN anomalies occurs at similar rates in the recently harvested vs stored accessions (**Figure 4C**). However, the nature of the detected errors appears to differ (not shown): in stored samples, RIN anomalies usually present as no-RIN-computed errors, which do not affect average RIN among replicates. In contrast, RIN anomalies in freshly harvested samples usually present as computed, but low, RIN values that are inconsistent with other replicates and lower the average RIN value when included.

**Figure 5:**
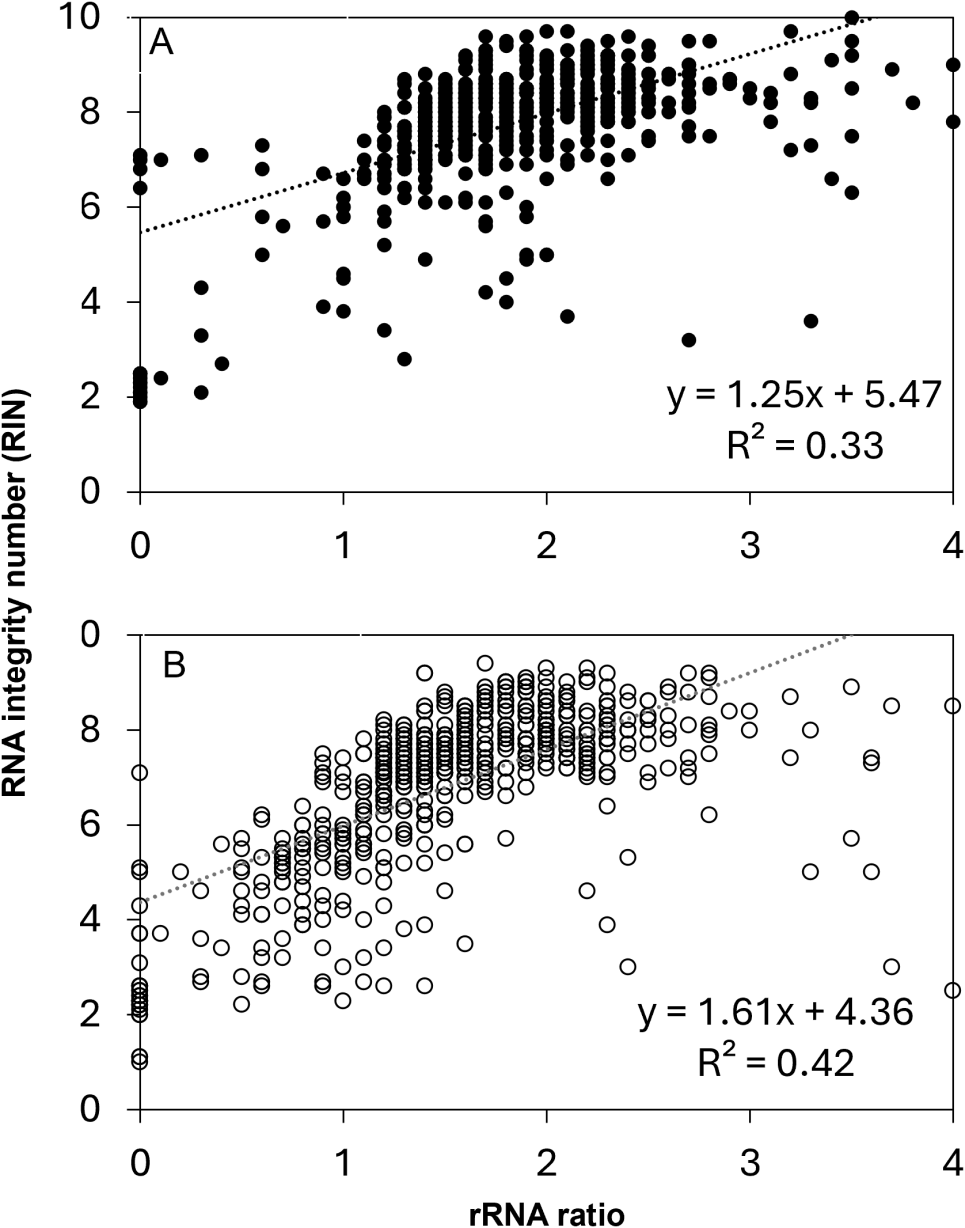
The relationship between RNA integrity number (RIN) and rRNA ratio in RNA samples from **(A)** recently harvested (closed circles) and **(B)** stored seed extracts (open circles). P < 0.0001 for both recently harvest and stored.

Because the RIN calculation is anchored by the prominence and position of rRNA peaks, we expect a strong correlation between rRNA ratio and RIN in this study set (**Figure 5**). Correlations for these relationships for non-anomalous RIN values and rRNA ratios < 4 were significant at P < 0.0001. The coefficients of the linear regressions vary for recently harvested and stored seeds, owing mostly to the greater number of points with rRNA ratio < 1 from stored seeds, as noted in **Figure 4**. RIN calculations are considered more reliable than rRNA ratios, especially in borderline cases when rRNA ratios <1 (values for RIN range from 1 to 7) or when rRNA ratio = 0 (a RIN value is sometimes calculatable) (**Figure 4B**).

### Do seed characteristics affect RNA extraction and characterization?

Our analysis of characteristics of RNA extracts that do and do not signify successful electrophoresis (previous section) revealed that low RNA concentration was an important factor. The mass of individual seeds varied considerably in this study (between 0.01 and >400 mg per seed with a median seed mass of 0.9 mg per seed). Samples containing few, tiny seeds or focusing on specific tissues with low RNA content are, therefore, at risk for providing unreliable RIN results. Moreover, variation of RNA quality among seed parts may confound assessment of its stability during dry storage. To address these questions, we assessed RNA yield and RIN for seed and seed parts in a cross section of species.

RNA content within seeds was approximated by dividing RNA yield by the number of seeds used in the extraction. RNA content ranged from 0.1 to >900 ng RNA/seed and averaged ~90 ng RNA/seed. RNA yield (RNA concentration (ng/µl)/sample mass (mg)) was significantly higher in seed parts with growing potential (i.e., the embryonic axis) than with tissues that served mostly as food reserves (i.e., nutritive tissues -endosperm or megagametophyte) for half of the species tested for this treatment, *Lupinus westianus* (p = 0.0203)*, Abies fraseri* (p = 0.0450)*, and Rhus kearneyi* (p = 0.0310) (**Table 2**). RIN was significantly higher in nutritive tissue compared to embryonic tissue for one species, *Amaranthus pumilus* (p = 0.0245; **Table 2**).

**Table 2:** Species used for the different treatments tested for differences in RNA quality.

Treatment	Species	Harvest year	% Empty	Seed mass (mg)	Cleaned					Not cleaned					Difference between treatments		[RNA]/mean sample mass	RIN
					Mean RNA sample mass (mg)	Mean [RNA] ng/µl	[RNA]/mean sample mass	Mean RIN	std dev RIN	Mean RNA sample mass (mg)	Mean [RNA] ng/µl	[RNA]/mean sample mass	Mean RIN	std dev RIN	RIN	std dev RIN	Pvalue	Pvalue
**Cleaned for empty seed**	*Sidalcea nelsoniana*	2021	36	2.583	16.4	679.7	41.5	8.2	0.2	19.2	478.8	25.0	8.3	0.3	-0.1	-0.1	**0.0319**	0.6532
	*Rhus kearneyi ssp. kearneyi*	2022	52	48.994	16.2	414.7	25.7	8.3	0.1	18.0	237.0	13.2	8.3	0.3	0.0	-0.1	**0.0025**	0.7698
	*Remirea maritima*	2021	53	0.404	5.9	2.8	0.5	3.4	N/A	5.1	4.8	0.9	3.2	N/A	0.2	N/A	N/A	N/A
	*Packera franciscana or Senecio franciscanus*	2022	94	0.313	13.9	115.8	8.3	2.4	N/A	9.6	195.7	20.4	2.0	N/A	0.4	N/A	N/A	N/A
	*Oxypolis canbyi*	2022	42	6.321	11.1	209.4	18.9	7.8	0.3	15.0	138.9	9.3	6.7	0.7	1.1	-0.4	0.1263	**0.0176**
	*Linum carteri* var. *carteri*	2021	69	0.351	8.1	39.5	4.9	8.2	N/A	6.4	41.4	6.4	2.3	N/A	5.9	N/A	N/A	N/A
	*Dubautia menziesii*	2021	87	0.806	18.6	194.5	10.5	8.2	0.9	34.5	65.2	1.9	7.6	0.1	0.6	0.9	0.1255	0.3467
	*Chrysopsis floridana*	2021	64	0.420	10.1	334.8	33.2	8.7	0.5	4.5	35.5	7.9	9.1	0.1	-0.4	0.4	**0.0071**	0.2411
	*Bidens torta*	2022	41	1.488	11.3	243.3	21.5	8.6	0.2	16.8	223.7	13.3	7.4	0.6	1.2	-0.4	0.1174	**0.0036**
	*Abies fraseri*	2023	48	7.608	9.5	145.7	15.4	7.8	1.3	23.1	16.1	0.7	2.9	0.8	5.0	0.4	**0.0033**	**0.0011**
	*Solidago plumosa*	2003	47	0.387	4.8	208.4	43.2	8.0	0.3	7.4	269.0	36.3	7.5	0.2	0.5	0.1	0.1090	**0.0224**
	*Remirea maritima*	2003	57	0.559	6.3	3.6	0.6	5.6	N/A	6.1	6.4	1.1	2.6	N/A	3.1	N/A	N/A	N/A
	*Lycium sandwicense*	2006	47	0.273	9.7	125.0	12.9	6.0	N/A	11.8	114.6	9.7	4.9	1.5	1.1	N/A	N/A	N/A
	*Echinocactus horizonthalonius* var. *nicholii*	1991	52	3.368	10.6	165.6	15.7	7.8	0.5	15.7	144.6	9.2	7.7	0.4	0.1	0.1	0.5531	0.0530
	*Cirsium pitcheri*	1991	49	9.837	11.6	325.5	28.2	8.3	0.6	15.3	234.7	15.3	8.2	0.3	0.1	0.3	**0.0330**	0.7157
	*Amaranthus pumilus*	1987	52	2.212	3.7	35.8	9.6	5.6	1.4	13.6	51.2	3.8	5.9	1.2	-0.2	0.2	0.1320	0.4911
**Seed coverings removed**	*Tephrosia angustissima* var. *corallicola*	2022	0	8.369	10.8	620.6	57.2	7.5	0.1	14.8	205.8	13.9	7.4	0.5	0.1	-0.3	**0.0001**	0.7908
	*Ornithostaphylos oppositifolia*	2022	17	4.028	11.0	39.7	3.6	8.7	N/A	10.1	155.7	15.5	8.6	0.1	0.1	N/A	N/A	N/A
	*Lomatium bradshawii*	2021	0	7.099	10.4	279.8	26.9	8.2	0.8	15.2	246.3	16.2	8.8	0.1	-0.6	0.7	**0.0245**	0.2137
	*Hibiscus dasycalyx*	2021	22	11.026	13.8	678.6	49.0	7.1	0.4	18.4	479.7	26.0	7.0	1.3	0.1	-0.9	**0.0151**	0.4494
	*Eryngium aristulatum* var. *parishii*	2022	27	0.710	10.7	235.7	22.0	8.4	N/A	9.5	144.8	15.2	8.6	0.3	-0.2	N/A	N/A	N/A
	*Eriogonum cusickii*	2022	4	1.127	13.2	48.0	3.6	8.5	0.6	15.8	231.1	14.6	8.4	0.2	0.1	0.4	0.0679	0.8336
	*Echinocactus horizonthalonius* var. *nicholii*	2022	14	6.948	11.1	36.4	3.3	7.3	0.5	13.5	50.3	3.7	7.7	1.4	-0.4	-0.8	0.7265	0.5151
	*Dodonaea viscosa*	2022	3	5.151	11.1	243.7	22.0	8.2	0.2	17.1	290.6	17.0	8.2	0.1	0.0	0.1	0.2723	0.8294
	*Cirsium pitcheri*	2021	21	5.664	9.2	226.1	24.6	8.8	0.3	15.2	235.5	15.5	9.1	0.4	-0.2	-0.1	0.2704	0.4220
	*Cimicifuga elata*	2022	0	2.150	11.7	137.4	11.7	7.9	0.2	15.5	254.3	16.4	7.9	0.2	0.0	0.0	**0.0345**	0.9650
	*Ceanothus cyaneus*	2021	26	2.206	17.1	133.8	7.8	8.5	0.1	34.9	230.4	6.6	8.1	0.2	0.4	0.0	0.7907	**0.0355**
	*Carex comosa*	2022	23	0.978	10.4	1.4	0.1	7.0	1.0	13.5	1.5	0.1	6.8	N/A	0.1		N/A	N/A
	*Astragalus linifolius or Astragalus rafaelensis*	2023	0	6.088	11.7	789.9	67.3	7.5	0.7	15.3	213.3	13.9	7.1	0.3	0.3	0.4	**0.0011**	0.5799
	*Astragalus bibullatus*	2021	0	5.978	9.8	558.3	57.0	6.9	0.3	16.2	62.3	3.8	7.1	0.2	-0.2	0.1	**0.0006**	0.3297
	*Argemone glauca*	2022	12	3.294	14.1	6.7	0.5	7.7	0.9	13.8	11.3	0.8	7.7	0.1	0.0	0.8	0.0617	0.4626
	*Arctostaphylos catalinae*	2023	12	89.592	11.9	18.2	1.5	8.2	0.8	56.6	18.8	0.3	8.0	0.9	0.2	-0.1	0.1326	0.7861
	*Amsonia tharpii*	2023	3	18.795	12.0	202.4	16.9	7.4	0.1	16.4	114.8	7.0	7.0	0.4	0.4	-0.3	0.2803	0.5555
	*Amelanchier nantucketensis*	2023	7	5.737	10.8	80.8	7.5	8.9	0.2	34.1	8.2	0.2	7.0	1.8	2.0	-1.6	**0.0060**	**0.0344**
	*Amaranthus pumilus*	2022	5	2.875	13.2	24.3	1.8	8.4	0.7	12.1	40.4	3.3	8.4	0.4	-0.1	0.3	**0.0360**	0.4438
	*Actaea arizonica*	2022	5	0.851	12.3	208.8	16.9	8.5	0.4	17.3	342.2	19.8	8.5	0.6	0.0	-0.2	0.3048	0.9855
	*Rhus kearneyi ssp. kearneyi*	1986	25	42.672	9.9	269.8	27.3	7.4	0.4	31.3	189.8	6.1	7.2	0.2	0.3	0.2	**0.0110**	0.8930
	*Penstemon clutei*	1991	15	0.430	19.5	18.9	1.0	2.8	N/A	17.5	31.4	1.8	2.6	0.0	0.2		N/A	N/A
	*Eutrema penlandii or Eutrema edwardsii*	1988	7	0.307	10.1	621.7	61.6	8.8	N/A	6.7	645.4	96.3	7.8	0.1	1.0		N/A	N/A
	*Eriogonum cusickii*	1983	15	1.024	13.0	62.3	4.8	6.3	0.3	16.2	61.5	3.8	4.2	1.1	2.0	-0.8	0.5776	0.7184
	*Dodonaea viscosa*	1990	0	5.482	10.4	208.4	20.0	6.1	0.7	15.9	319.8	20.1	5.0	2.2	1.1	-1.4	0.9843	0.3009
	*Astragalus magdalenae* var. *peirsonii*	2003	0	20.416	9.9	937.8	94.3	6.9	0.5	31.3	73.2	2.3	7.6	N/A	-0.7		N/A	N/A
	*Astragalus albens*	1995	0	2.352	10.1	495.3	49.2	8.5	0.6	13.9	283.2	20.4	7.8	0.1	0.7	0.5	**0.0357**	0.1514
	*Abronia umbellata* var. *breviflora*	1990	1	13.457	16.2	292.2	18.0	7.8	0.4	15.4	77.9	5.1	7.4	0.3	0.4	0.0	0.1664	0.0970
					Mature					Immature					Difference between treatments			
Maturity	*Amorpha herbacea* var. *crenulata*	2021	0	3.712	4.7	274.7	58.9	7.0	0.0	4.2	236.8	56.8	6.7	0.3	0.3	0.3	0.7600	0.1318
	*Physaria obcordata*	1987	0	3.933	5.5	180.3	32.9	4.3	2.4	6.8	168.1	24.8	6.8	0.0	-2.5	-2.4	0.4608	**0.0182**
	*Geum geniculatum*	2009	19	1.276	18.9	136.8	7.2	7.3	0.2	12.2	80.8	6.6	6.1	1.0	1.2	0.7	0.9454	**0.0486**
	*Astragalus tyghensis*	2000	3	4.061	10.8	434.4	40.1	7.6	0.2	9.0	409.1	45.4	7.4	0.3	0.2	0.1	0.3681	0.1866
					Embryo					Nutritive Tissue					Difference between treatments			
Tissue type	*Rhus kearneyi ssp. kearneyi*	2022	52	48.994	7.2	173.5	24.0	8.2	0.1	20.8	403.9	19.4	8.5	0.2	-0.3	-0.1	0.1513	0.4534
	*Lupinus westianus* var. *aridorum*	2021	0	16.152	2.1	171.2	80.2	7.4	0.2	10.2	368.2	36.2	7.3	0.6	0.0	-0.4	**0.0203**	0.4929
	*Astragalus magdalenae* var. *peirsonii*	2021	0	22.111	3.6	288.3	80.1	8.1	0.5	17.3	927.3	53.5	7.5	0.2	0.7	0.2	0.2522	0.0723
	*Abies fraseri*	2023	48	7.608	2.1	144.5	69.6	8.0	1.3	10.2	83.6	8.2	7.2	2.5	0.9	-1.2	**0.0450**	0.4838
	*Rhus kearneyi ssp. kearneyi*	1986	25	42.672	1.5	86.5	59.0	7.7	0.4	13.5	199.0	14.7	7.7	0.3	0.0	0.0	**0.0310**	0.4427
	*Amaranthus pumilus*	1987	52	2.212	2.0	52.9	26.4	6.6	0.4	6.3	10.3	1.6	4.3	1.3	2.3	-0.9	0.0682	**0.0245**
	*Abies fraseri*	2006	95	5.879	1.4	10.0	7.1	2.5	N/A	6.1	9.9	1.6	N/A	0.5			N/A	N/A

Percent (%) empty, indicates the amount of seed that was determined empty in a given seed lot for that harvest year. Bold values indicate a significant difference within species for the given treatment.

Many of the seeds in the study had thick outer-coverings, likely remnants of degraded maternal tissues. The presence of the thick seed coat reduced RNA yield in 10 of the 28 accessions studied and RIN was lower in 2 of the 28 accessions studied (**Table 2**). The two accessions that had a lower average RIN when seed coverings remained on the seeds prior to extraction were *Amelanchier nantucketensis* (p = 0.0344; 70% mass removed) and *Ceanthus cyaneus* (p = 0.0355; 3% mass removed).

Seed filling was tested to determine if extracting RNA from a sample that was cleaned for empty seed versus a sample that bulked filled and empty seed impacted the overall average RIN for a given species. There were 15 species that had greater than 36% empty seed in a sample (**Table 2**), with an overall average of 56% empty seed. Of 10 samples tested statistically, four had higher RIN in the cleaned rather than the bulked sample (*Abies fraseri* 2023H (p = 0.0011), *Bidens torta* 2022H (p = 0.0036), *Oxypolis canbyi* 2022H (p = 0.0176) and *Solidago plumosa* 2003H (p = 0.0224)). An additional five samples were not tested statistically, due to low seed numbers, but suggested an impact of empty seeds on RIN. RNA yield but not RIN was lower in 5 of the remaining 6 samples (**Table 2**). This extra step of cleaning seed had mixed effects on RIN values. For example, cleaned and uncleaned samples of recently harvested seeds of *Abies fraseri* (95% empty) provided average RIN values of 7.9 and 2.9, respectively. While the same procedure in recently harvested seeds of *Dubautia menziesii* (89% empty) presented RIN values of 8.2 and 7.6 for cleaned and uncleaned samples, respectively.

Homogeneity in seed maturity is also difficult to control in seeds collected from wild populations. There were four species with mixtures of brown (mature) and green (immature) seeds in a given sample collection. When RNA was extracted and characterized for the two phenotypes separately, there was no evidence of an effect on RNA yield, but half of these taxa had significant differences in RIN average (**Table 2**). *Geum geniculatum* 2009H, had a slightly lower average RIN for immature seed than mature seed (p = 0.0486) and *Physaria obcordata* 1987H had a higher RIN for immature seeds (p = 0.0182.)

## Discussion

We examined a laboratory test that detects aging in seeds, particularly seeds from wild populations. Traditional germination tests are widely used by genebanks and seed companies for crop seeds, for which germination behavior is well characterized and amenable for testing. Numerous features of non-domesticated seeds complicate seed testing. For example, germination cues are not known, seeds are difficult to acquire and in low supply, test results are frequently inconsistent due to uncontrollable features of the seed and asynchronous germination. In this study, we demonstrated that assaying RNA integrity is a promising new test of seed quality that is quantitative, standardizable, sensitive to time-dependent changes and adaptable to future automation. We evaluated RNA integrity number (RIN) between pairs of seeds from over 100 wild, endangered species. Seeds from one cohort were harvested recently (2022 ± 1yr) and seeds from the older cohort were harvested at least 16 years ago (1995 ± 6 years) and stored in a genebank (mostly at -18°C, but a few at 5°C and a few cryogenically). We used standardized techniques and commercially available kits to assess RIN from all groups. With few exceptions, RIN values among species in the recently harvested cohort were consistently high, despite high diversity among accessions. In contrast, there was wider variation and lower RIN values measured in the stored cohorts, indicating that time-dependent fragmentation had occurred in dry seeds.

In general, there was sufficient RNA within dry seeds to analyze for RIN. An RNA concentration of 25 ng RNA µl^-1^ or more is recommended for reliable RNA characterization (manufacturer’s protocol). This was easily obtained for most seeds using a sample mass of 9 to 12 mg. Variation in RNA yield was broad, due to uncontrolled factors during extraction as well as seed traits. We noted that samples smaller than 9 mg yielded more than sufficient RNA in about ¼ of the species, suggesting that we, perhaps, used more seeds than needed to glean reliable RIN values. We also noted that RNA yields were consistently low in some seeds, for example, seeds from Ericaceae and Cyperaceae tended to have lower RNA yields compared to other species; sample sizes between 15 to 20 mg per replicate may be preferable for seeds in these families. For tiny seeds, (seed mass < 0.08 mg seed^-1^; e.g., *Leiophyllum buxifolium* (formerly *Kalmia buxifolia*) and *Vaccinium crassifolium* ssp. *sempervirens* (Ericaceae) or *Cyanea angustifolia* and *Clermontia kakeana* (Campanulaceae)), total mass of the accession was small, and we needed to either reduce the standard 9-12 mg sample size for each replicate or reduce the number of replications, or both. RNA extractions from seeds within Campanulaceae appeared to yield reliable RIN values despite small sample size and low replication; however, data produced for seeds within Ericaceae tended to be problematic (discussed below). More than 100 seeds were needed per replicate for some species producing tiny seeds. The number of seeds used for a RIN determination declines as seed mass increases, and seed consumption for RIN and standard germination tests are both about 50 seeds when seed mass is about 0.8 mg seed^-1^. Genebanks are deeply concerned about depleting accessions by testing needed to meet curatorial standards (FAO, 2018).

RNA quality was usually high in recently harvested seeds from the 103 wild species we studied. Average RIN values ranged between 7 and 9 for 88 species (**Table 1**), which is slightly higher than RIN reported for recently harvested commercial seed lots (Fleming et al., 2017, 2019). Some of that difference may be explained by seed collectors’ speed and care when processing seeds from endangered species. Average RIN was less than 5 for 4 species from the recently harvested cohort (*Packera franciscana*, *Polemonium occidentale* ssp. *lacustre*, *Remirea maritima*, *Remya kauaiensis*), all of which had relatively high proportions of unfilled seeds (> 50% empty) as well as relatively small seeds (< 0.4 mg seed^-1^) that precluded dissection – an extra step we took for larger seeds to enrich the filled-seed fraction. High incidence of empty seeds (e.g., *Dicerandra immaculata* (80% empty); *Lycium sandwicense* (30% empty) and/or low yielding RNA (Cyperaceae) explain some of the moderate RIN values (between 5 and 7) in the remaining species of the recently harvested cohort.

RIN values that are lower (or higher) than expected present questions of the reliability of the assay. The more common application of RIN assessments – to alert when an extraction yields RNA too degraded to sequence (Mueller et al., 2004; Schroeder et al., 2006)– suggests that experimental errors during extraction and electrophoresis might bias interpretation of the extent of RNA fragmentation *in planta*. We found that simply repeating the electrophoresis run using archived extracts resolved most problems with missing data or ambiguities, while not consuming more seeds. Our initial error rate of about 7% (of 1365 runs) was reduced to about 3% by a second electrophoresis of the extract that yielded suspect data initially. The incidence of RIN errors was significantly higher in samples containing low RNA concentration (< 30 ng µl^-1^). RIN errors were also higher in samples with low rRNA ratio (<1). Extracts that had both low RNA concentration and low rRNA ratio accounted for 71% of RIN errors. Experimental errors occur at the same frequency in both recently harvested and stored accessions, and so we do not anticipate these to be influential in assessing the effects of storage. Overall, flawed extractions affecting average RIN occurred in about 0.5% of the RIN measurements presented in the study.

Aside from low RNA concentration, we found few other factors that consistently affected the reliability of RIN measurements. Standardized procedures, with few adjustments for varying seed traits, led to few anomalies or ambiguities in the data and attest to broad applicability of using RIN across diverse seeds that vary by phylogeny, morphology, composition and germination behavior. In contrast, germination assays require conditions tailored for individual species (Hay and Probert, 2013); often the optimal conditions are unknown for seeds of wild species, leading to ambiguous calls on seed quality. Collectors do their best to optimize harvest times; even still, some samples may include seeds at various levels of maturity (Hay and Probert, 1995). This study demonstrated that tissue type or prevalence, including bulky or water-impermeable seed coverings and embryonic axis vs nutritive tissues may affect RNA concentration in some species but not RIN outcomes (**Table 2**). This conclusion is consistent with previous reports using legume crop seeds, (Fleming et al., 2017, 2019; Walters et al., 2020) and provides reassurances that seed dissections are rarely needed.

The general insensitivity of RIN to the presence of bulky, low RNA-containing tissues might make it an unreliable test for seed fill. In accessions with high prevalence of empty seeds, we found that additional steps to remove seeds lacking nutritive tissue or embryo tended to increase RNA yield. As described earlier, low RIN was observed in some recently harvested accessions with low seed fill and seeds too small for dissection (e.g., *Packera franciscana*, *Polemonium occidentale* ssp. *lacustre*, *Remirea maritima*, *Remya kauaiensis*); however, the opposite was also encountered (e.g., *Castilleja kaibabensis*, *Chenopodium oahuense*, and *Chrysopsis floridana*). The inconsistent effect of unfilled seeds on RIN may be related to the presence/absence of residual seed tissues with degraded RNA in under-developed embryos. It will be difficult to conduct paired comparisons of RIN among populations or cohorts without accounting for how seed fill affects RIN.

Of all the variables tested to determine potential influence on RIN, the difference among cohorts was the most clear and consistent (p = 0.0037). Difference of average RIN between recently harvested and stored cohorts was greater than 1 in 42 of the 101 species, and less than -1 for just 5 species, of which 4 had confounding problems of seed fill (e.g., *Packera franciscana*, *Polemonium occidentale* ssp. *lacustre*, *Remirea maritima*, *Remya kauaiensis*). In a previous paper, we showed that a RIN difference of about 0.5 is needed to establish significance with 6 replicates (Tetreault et al., 2023). Actual significance of RIN differences between cohorts will be presented in a subsequent paper that also compares germination results (Walters et al. *in progress*). The comparisons attempt to detect aging in stored seeds and this requires the assumption that RIN values for the recently harvested cohorts are representative of initial RIN values for the stored cohorts (RIN technology was not available in the 1990s when most of the stored cohorts were harvested). That assumption appears safe considering the great efforts to collect from the same populations and process the seeds in the same way, as well as the high quality of seeds obtained (with a few notable exceptions) and the consistency of RIN in the recently harvested seeds.

This work provides important confirmatory evidence that RNA quality declines with storage time and shows the applicability of the assay for a large and diverse set of seeds from wild populations native to the US. Demonstrating that RNA degradation is a marker for seed aging is challenging. Aging is currently measured in seeds as increasing mortality after an initial period of no viability change. In other words, aging in seeds is defined by lethal effects, not by the accumulation of nonlethal damage. RIN is likely to reflect non-lethal damage, and because it declines linearly with time (Fleming et al., 2017, 2019; Tetreault et al., 2023) it can be detected before viability changes are noted. Thus, RIN cannot be used as a viability assessment per se and in fact, we expect a poor correlation of RIN and viability. However, detecting degradation before mortality can be powerful if the rate of degradation (i.e., the slope of a linear time course) correlates with the *duration* that the seed stays viable (i.e., longevity). Hence, developing this work further requires continued monitoring of seed quality using both germination and RIN assays.

### Summary

Genebanking seeds collected from wild species is an important conservation strategy that maintains viable, genetically representative germplasm for restoration or research. A key challenge is maintaining viability over decades, especially when there are no guidelines for survival duration. To address this major unknown, most genebanks monitor viability periodically using germination tests (Hay and Probert, 2013; van Treuren et al., 2013; Solberg et al., 2020). Germination tests of wild seeds can be especially time-consuming and yield ambiguous results if germination protocols are not available. Moreover, germination tests provide a snapshot of the seed quality and do not provide insights for an expiration (or best used by) date. Thus, genebanks are consigned to test at frequencies that may be too broad and miss detection of a viability threshold, or too narrow and unnecessarily deplete a valuable seed sample.

While most of the work to-date exploring RIN as a marker for seed aging uses commercial seed lots, here we show that RIN assessments may be uniquely amenable for quality assessments of seeds from wild populations. First and foremost, RIN may provide information that can be translated to seed longevity, which will help optimize quality monitoring frequencies as well as to estimate when seed accessions should be used or regenerated. A complete RIN assessment can be completed within a week, while germination assays for wild seeds can take 6 months or more (when a protocol is available). RIN procedures are highly standardized (using purchased kits), despite wide variation among species, and results are highly reliable, especially electrophoresis can be repeated on samples if data appear anomalous. Germination tests provide the “gold standard” assessment on how to grow out valuable germplasm, and seedlings resulting from a germination test can be re-purposed if the need is dire. The value of quality assessments in genebanked samples is generally recognized, but the cost of consuming valuable seeds by testing is lamented. Plant fecundity and seed size are important factors in optimizing curation strategies to ensure genebanking goals are met.

## Data Availability

The raw data supporting the conclusions of this article will be made available by the authors, without undue reservation.

## Supplementary material

Supplementary Figure 1 Immature and mature seed for **(A)** *Astragalus tyghensis and* **(B)** *Geum geniculatum.*
