# Supplementary figures and images for: Assessing the RNA integrity in dry seeds collected from diverse endangered species native to the USA
Source: Front Plant Sci. 2025 May 13;16:1585631. doi: 10.3389/fpls.2025.1585631 (PMC12106311; doi:10.3389/fpls.2025.1585631)

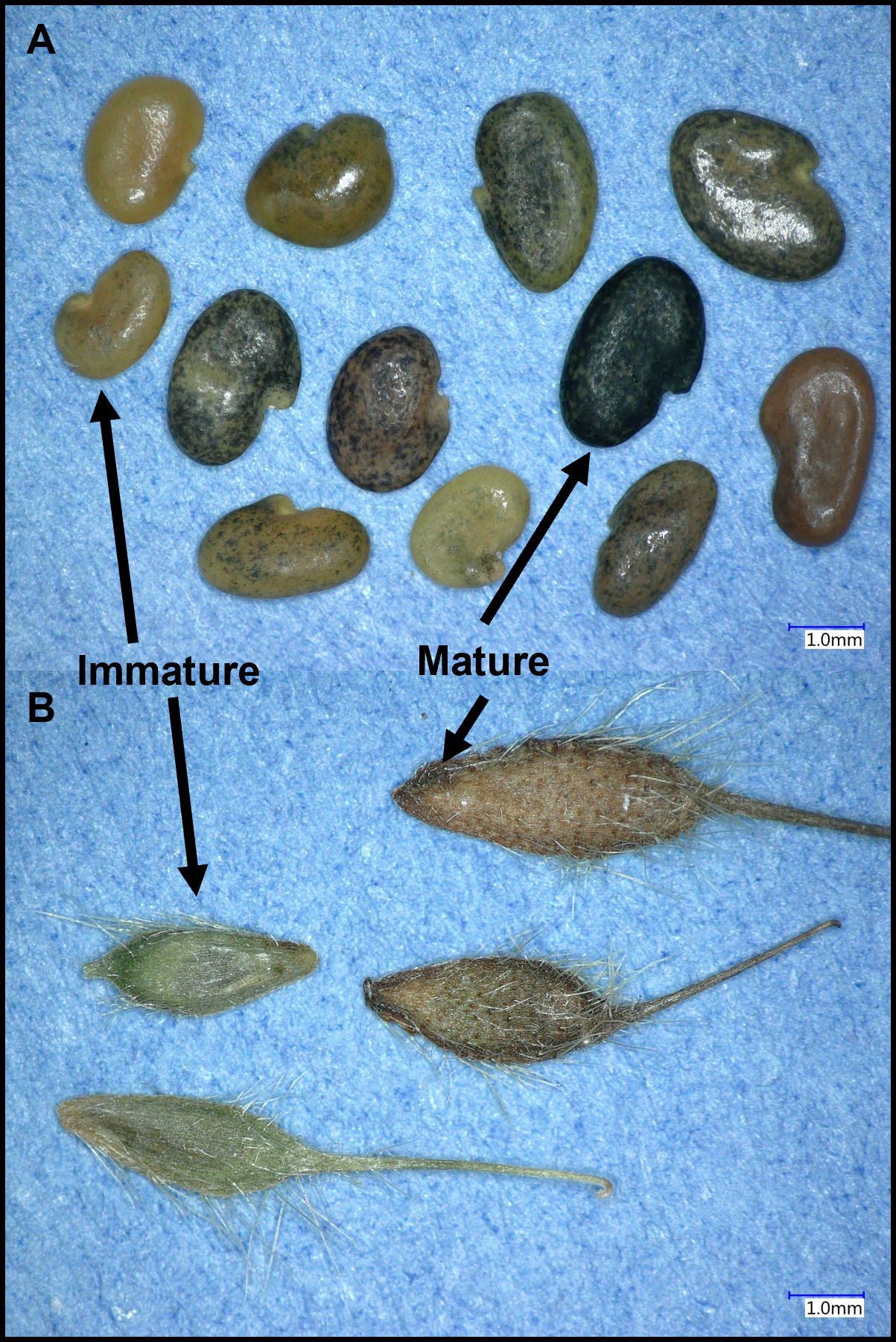

Supplement: Supplementary Figure 1 — Immature and mature seed for (A) Astragalus tyghensis and (B) Geum geniculatum. [file Image1.tif]
